# Feasibility, use and usability of an adapted mHealth tool for dementia risk reduction among middle-aged and older adults in Germany: protocol for a non-randomised pilot study

**DOI:** 10.1136/bmjopen-2026-117886

**Published:** 2026-09-16

**Authors:** Andrea E Zülke, Melanie Luppa, Kay Deckers, Sebastian Köhler, Steffi G Riedel-Heller

**Affiliations:** 1 Institute of Social Medicine, Occupational Health and Public Health, University of Leipzig, Faculty of Medicine, Leipzig, Germany; 2 Alzheimer Centrum Limburg, Department of Psychiatry and Neuropsychology, Mental Health and Neuroscience Research Institute (MHeNs), Maastricht University, Maastricht, The Netherlands

**Keywords:** Telemedicine, Cognition, Dementia, Primary Prevention

## Abstract

**Introduction:**

Several potentially modifiable factors are associated with the risk of cognitive decline and dementia, indicating potential for risk reduction via lifestyle modifications. While multidomain interventions targeting lifestyle changes for dementia risk reduction are often highly selective and resource-intensive, eHealth/mHealth interventions offer a potentially low-cost, scalable approach to promote brain-healthy lifestyles for a large number of individuals. This protocol outlines a pilot study to test an mHealth intervention for dementia risk reduction among middle-aged to older adults in Germany.

**Methods and analysis:**

We will translate and adapt the smartphone-based intervention MyBraincoach for dementia risk reduction, developed at Maastricht University in the Netherlands, to the German healthcare setting and test the app in a 12-week, non-randomised pre-post pilot study. Key feasibility outcomes include feasibility and success of recruitment, use and usability of the app. Exploratory outcomes include changes in dementia risk profiles, dementia literacy, health-related quality of life, self-efficacy and motivation for behaviour change. A sample of n=75 adults aged 40–75 years will be recruited across Germany, using diversified convenience sampling. Data will be collected using web-based questionnaires at baseline and follow-up after 12 weeks. Feasibility and success of recruitment, including recruitment rates and sample characteristics, will be evaluated descriptively. Changes in dementia risk profiles and other secondary outcomes will be investigated using dependent-samples t-tests and McNemar’s tests as appropriate. Lastly, a moderated remote focus group will be conducted with n=8 participants of the pilot study to gain insights on the target group’s experiences with usage of the app and to assess suggestions for modification and improvement. Respective data will be analysed using structured content analysis.

**Ethics and dissemination:**

Ethical approval was obtained from the Ethics Committee of the Medical Faculty of Leipzig University (ref.: 476/22-ek). Findings will be disseminated via peer-reviewed publications, presentation at (inter)national scientific conferences and social media channels, respectively. Results will inform design and sample size of a confirmatory randomised controlled trial to assess effectiveness of the intervention.

**Trial registration number:**

German Clinical Trials Register (DRKS00038349).

**Strengths and limitations of this study**

- Internet-based interventions constitute an innovative, accessible approach for delivering tailored advice on brain health, possibly able to reach large parts of society and allowing for large-scale implementation.
- We will test an evidence-based mHealth tool in German adults aged 40–75 years, targeting dementia risk reduction and brain health based on a validated dementia risk score.
- Using both quantitative and qualitative methods, we will provide insights on the feasibility of recruitment, use, usage patterns and usefulness of the app, as well as users’ perspectives, enhancing involvement of the target group.
- Recruiting diverse samples, including, for example, adults with lower socioeconomic backgrounds, is challenging in lifestyle interventions, and use of eHealth tools is generally lower in socioeconomically disadvantaged populations, complicating inclusive study sampling.
- Participants will be included irrespective of their baseline dementia risk to enhance the applicability of the findings to the general population, which may limit detectable changes in dementia risk profiles due to potential floor effects.

## Introduction

 The number of people living with dementia is increasing globally, particularly due to population ageing, with cases expected to triple from an estimated 55 million in 2019 to 152 million in 2050.^1^ Despite recent advances in disease-modifying treatments,^2 3^ their clinical indication is narrow and a readily available cure is currently not on the horizon. A vast amount of research indicates possibilities for dementia risk reduction by modification of risk factors. Established risk factors include, for example, hypertension, diabetes mellitus, physical inactivity, hearing loss, social isolation, smoking or depression.^4^ Further evidence is accumulating for other individual-level and population-level risk and protective factors.^5^

Evidence on lifestyle-based risk factors for dementia has resulted in the launch of numerous randomised controlled trials (RCTs), assessing the effects of lifestyle modifications on cognitive performance and further outcomes, for example, dementia risk scores, in at-risk populations. Particularly, multidomain interventions targeting several risk factors simultaneously have been suggested as promising. The Finnish Geriatric Intervention Study to Prevent Cognitive Decline and Disability (FINGER) reported small but significant effects of an intensive 2-year multidomain lifestyle intervention on cognitive performance in older (60–77 years) adults at increased risk for dementia.^6^ Currently, several studies, organised in the World-Wide-FINGERS-network, are adapting this approach to different regional and cultural settings as well as target populations globally.^7^ Findings of completed trials are mixed, with some interventions showing positive effects on cognitive performance,^8–10^ while others report negative results.^11–13^ However, respective interventions are often resource-intensive and highly selective, targeting older high-risk populations rather late in life (>70 years), therefore benefiting only small parts of society. Evidence suggests that the adverse effects of risk factors on cognitive function seem to increase with ageing,^14^ and many modifiable risk factors for dementia are already highly prevalent among younger populations (40–60 years).^15^ Calls are being made to address dementia risk reduction and brain health earlier in life, using scalable, accessible means to maximise effects on a population level.^16 17^

The recently updated WHO guidelines on risk reduction of cognitive decline and dementia emphasise the potential of tailored multidomain interventions across the life course, targeting several risk factors for dementia simultaneously. This includes interventions targeting depression, sleep, hearing loss and social isolation. The WHO further emphasises the need for culturally tailored and context-specific interventions, delivered in-person or digitally, to increase accessibility and reach.^18^ To date, implementation research addressing feasibility and scalability of respective interventions remains limited.

Internet-based (eHealth) or mobile (mHealth) interventions have been highlighted as an innovative, accessible and potentially cost-effective approach to provide tailored advice on brain health, allowing for large-scale implementation of highly individualised interventions. Respective interventions might further be useful to raise awareness about brain health, as evidence suggests that knowledge of several risk factors for cognitive decline and dementia, particularly cardiovascular and metabolic risk factors, is limited among the general population.^19–24^ A meta-analysis reported small to moderate effects of eHealth interventions on cognitive performance and dementia risk scores.^25^ Recent findings from the Australian Maintain Your Brain-trial further reported beneficial effects of a personalised digital intervention on cognitive performance, a validated dementia risk score and several lifestyle factors, including diet, physical activity and psychological distress at 3-year follow-up.^9^ Several respective eHealth/mHealth interventions for primary or secondary prevention of dementia, daily life support, screening and decision making have been developed and evaluated recently, aiming to support primary or secondary prevention, screening, decision making or support in daily life, with most interventions in the stage of early evaluation and maturity.^26^ Dementia risk reduction using eHealth/mHealth interventions in primary care/general practitioner (GP) settings have proven feasible in several studies,^27 28^ further suggesting potential for implementation on a scalable level. However, it remains unclear whether a culturally tailored multidomain mHealth intervention can be successfully implemented and accepted in a German population.

Against this background, we aim to test a translated and adapted version of an evidence-based mHealth intervention for dementia risk reduction, the MyBraincoach-app,^29^ in a pre-post pilot study among German adults aged 40–75 years. Feasibility and acceptability will be evaluated in terms of:

- Feasibility of recruitment.
- Success rate of retention of participants at 12 weeks follow-up assessment.
- Usage (patterns) of the app.
- Perceived use and usability of the app.
- Reasons for non-use and study dropout.

Further exploratory objectives are:

- To investigate change in knowledge of risk and protective factors for dementia.
- To describe changes in dementia risk profiles at 12 weeks follow-up.
- To assess changes in self-efficacy, motivation for behaviour change and health-related quality of life.
- To gain exploratory insights into participants’ perception of the app by means of a moderated focus group.

A proof-of-concept-study in community-dwelling middle-aged adults in the Netherlands investigated usage patterns and appreciation of the Dutch app (V.1.0), as well as changes in knowledge and attitudes towards dementia risk reduction.^29^ While overall app use was modest (56%), those who used the app evaluated it positively, increased their knowledge on dementia risk reduction and showed more positive beliefs about health behaviour change at follow-up. In a further proof-of-concept-study testing the app in Dutch primary care patients (n=188), 69.2% of users considered the app useful.^28^ The app improved knowledge on dementia risk reduction, while dementia risk improved in both the intervention (GP consultation+app) and control group (GP consultation only).

The present study aims to provide context-specific evidence on recruitment and retention, patterns of app engagement, perceived usefulness and usability and accessibility of an adapted version of the app in a German setting. By combining a quantitative feasibility assessment with qualitative user feedback, it will provide implementation knowledge required to refine the intervention and inform the design of a subsequent RCT.

## Methods and analysis

### Study design

We will conduct a non-randomised pre-post pilot study to test the translated and adapted MyBraincoach-app among n=75 middle-aged and older adults in Germany. The pilot study will be conducted in accordance with the extended Consolidated Standards of Reporting Trials (CONSORT) statement for feasibility and pilot trials.^30^ Further, to gain a better understanding of user experiences and potential suggestions for improvement, a moderated, video-based focus group will be conducted with n=8 participants following completion of the pilot study. These findings will inform the decision to conduct a subsequent confirmatory RCT to investigate intervention effectiveness.

### Participants

Eligible for the study will be individuals aged 40–75 years, recruited nationwide in Germany, with internet access by use of a smartphone with internet connection and sufficient command of the German language. Recruitment is planned to commence on 1 November 2026 and, following rolling enrolment and individual 12-week intervention periods, is anticipated to be completed after the final follow-up assessment and focus group by 30 June 2027. Individuals with a dementia diagnosis or severe conditions possibly interfering with safe use of the app will be excluded from participation. Participants will provide information on current diagnoses using a custom self-report questionnaire. Further exclusion criteria include current participation in other studies testing healthy ageing interventions. No minimum number of modifiable risk factors will be required for study participation, and individuals with few or no risk factors will remain eligible, as the intervention also aims at improving knowledge about the links between lifestyle and brain health and reinforce the maintenance of protective health behaviours.

### Recruitment

Participants will be recruited primarily online using convenience sampling. To mitigate the risk of recruiting a predominantly highly educated and health-literate sample, a broad and diversified recruitment strategy will be employed. Recruitment channels will include advertisements in health-related and general-interest online forums and magazines, community newsletters, volunteer registries, social media channels of Leipzig University and other research institutions or non-governmental organizations, as well as community health centres and GP practices. Additional recruitment efforts will target platforms and settings with broader sociodemographic reach. Interested individuals will click on a link leading to the study homepage, where they will be able to sign an online informed consent form and provide their contact information. Eligible participants will then receive a personalised QR-code via email to download the app to avoid non-participants downloading the app, as well as a link to the baseline assessment to complete online. Questionnaire data and app metadata will be linked via a unique pseudonymised study ID embedded in the personalised survey links and stored in the app at first login. Participants will be reimbursed with 20€ for their efforts.

### Study procedures

After signing the informed consent form, participants will complete the baseline assessment, comprising standardised questionnaires on sociodemographic factors, health-related quality of life, self-efficacy, motivation for behaviour change and knowledge about risk/protective factors for dementia (see ‘Outcomes and measures’ section). A reminder will be sent per email to those having consented but did not complete the baseline assessment after 2 weeks. If no baseline data are submitted after this reminder, only the signed consent and minimal contact information will be retained for documentation purposes, and no other personal data will be processed. Consenting participants will then be able to download the MyBraincoach-app. All participants will receive a reminder via email 2 weeks after enrolment in the study to encourage download and use of the app.

At the end of the intervention period (12 weeks), all participants will receive an email with a link to the follow-up assessment, containing the same questionnaires as used at baseline, as well as assessing perceived use and usability of the app. During the follow-up assessment, participants can provide consent to be re-contacted for participation in the video-based focus group. A remote focus group with an expected duration of 90–120 min will then be scheduled, including n=8 pilot study participants, moderated by a researcher experienced in qualitative methodology. Participants will be purposively selected from those consenting to be re-contacted. Subject to participant availability, selection will aim to include individuals with different levels of app engagement, varied educational and sex backgrounds.

### Intervention

The intervention to be tested is the MyBraincoach-app, which was developed and evaluated at the Alzheimer Centre Limburg, Mental Health and Neuroscience Research Institute at Maastricht University (Maastricht, the Netherlands) under the name ‘MijnBreincoach’. The app aims to improve knowledge of brain health and motivation for behaviour change in the general population through tailored lifestyle advice. It has been approved as an evidence-based tool by the Dutch municipal health services and the Ministry of Health, Welfare and Sport.

The central component and scientific basis of the app is the LIfestyle for BRAin Health (LIBRA)-score,^31^ capturing an individual’s potential for dementia risk reduction. It has been developed by researchers at Maastricht University, based on a systematic review and Delphi consensus study. LIBRA is a comprehensive modifiable dementia risk score, which has been extensively validated as a predictor of cognitive decline, incident dementia and biomarkers of brain health.^32–35^ Originally, LIBRA comprised 12 modifiable risk/protective factors for dementia, that is, diabetes mellitus, hypercholesterolaemia, chronic kidney disease, heart disease, obesity, hypertension, depression, physical inactivity, healthy diet, high alcohol consumption, smoking and high cognitive activity. Each factor is assigned a weight, based on the relative risk of said factor,^31^ resulting in a LIBRA-score ranging from −5.9 to +12.7 points. Higher scores indicate higher risk for dementia or more room for improvement through lifestyle modification. Following an update in 2024, based on an umbrella review of systematic reviews and a Delphi consensus study, three new risk factors were added, that is, sleep disturbances, low social contact and hearing impairment,^36^ with scores of the updated LIBRA ranging from −6.1 to 25.7 points. Respective app modules targeting said factors have been developed and implemented in the Dutch original version of the app at Maastricht University.

Participants will be advised to use the app independently on a daily basis for 12 consecutive weeks. Use of the app comprises the following steps:

- Completion of the LIBRA-assessment to identify present/absent risk factors for dementia (LIBRA profile), based on n=15 modifiable risk/protective factors for dementia. The LIBRA profile comprises three parts: ‘keep this up’, that is, sum of risk factors absent for the individual; ‘remember to manage well’, that is, sum of risk factors which are not directly amendable to change, for example, diabetes mellitus, but should be monitored regularly, for example, during usual visits to the attending GP; ‘room for improvement’, that is, sum of risk factors present for the individual and modifiable through lifestyle change. Preferably, participants will select a factor indicating room for improvement. However, participants with limited or no room for improvement may freely choose any lifestyle factor or health condition covered by the app.
- Choosing a lifestyle factor/condition to address, preferably among lifestyle factors indicating room for improvement. Users can change their risk factor of interest any time.
- Receipt of daily notifications for 14 consecutive days on the chosen lifestyle factor/condition; if a notification is not opened, its content is stored until the next day, that is, users can engage with a topic for more than 14 days if not all notifications are opened on a daily basis.
- Choice of a new lifestyle factor/condition to address.

The MyBraincoach app is designed to support users in behaviour change for dementia risk reduction by providing tailored advice based on participants’ individual risk profile, using micro-teaching (2–5 min reading time) notifications sent to users’ smartphones. Notifications can contain educational material, quizzes, personalised tasks/challenges, for example, setting specific goals for the chosen behaviour. The intervention is based on the theory of planned behaviour,^37^ which has shown very good predictive qualities for health behaviour change, control beliefs and attitudes in studies on prevention and public health approaches.^38^ Following this theory, an intention to perform specific behaviours is a function of the underlying attitudes toward the behaviour, subjective norms and perceived behaviour control. The respective app modules aim to positively influence attitudes and control beliefs towards behaviour change (eg, increased physical activity, smoking cessation) to evoke small steps for lifestyle changes beneficial for brain health by providing personalised feedback on modifiable risk and protective factors for dementia. In a study testing the Dutch version of the app, users evaluated MyBraincoach positively, with 75.5% stating that they enjoyed using the app, and 80.4% indicating that the app improved their understanding of the concept of brain health.^29^ Further, in a proof-of-concept study in Dutch primary care patients, 69.2% of users considered the app useful, and the app improved participants’ knowledge on dementia risk reduction. Dementia risk, assessed using the LIBRA-score, improved in both the intervention (GP consultation+app) and control group (GP consultation only^28^).

Uptake and use of eHealth tools is often lower in individuals with lower levels of education.^39 40^ Therefore, we will conduct usability testing sessions, including individuals with varying degrees of education to ensure usability across different educational backgrounds prior to the pilot study. Research on eHealth use, specifically for health promotion and prevention, in people with disabilities is currently scarce. Single studies identified higher perceived difficulty and overall lower eHealth use in people with disabilities than in non-disabled controls.^41^ To increase accessibility, app contents will be checked against the Web Content Accessibility Guidelines. The app will be tested for readability with a screen reader and usability testing sessions with individuals with visual impairment will be conducted prior to the pilot study. Usability testers (n=6) will be recruited using convenience sampling (eg, advertisements at community centres, religious/cultural organisations, health and social services, social media channels of Leipzig University, personal networks) and will be invited to test the app during a testing session (scheduled duration: ca. 90 min) at Leipzig University. Participants will report their experience with use of the app directly to a member of the research team.

### Outcomes and measures

As this pilot study is not designed to assess intervention effectiveness, no single primary endpoint was specified. The primary objective is to assess the feasibility and acceptability of the adapted MyBraincoach app and the associated study procedures. No single primary endpoint was specified, as the study is not designed to assess intervention effectiveness. Key feasibility outcomes include feasibility of recruitment, success rate of retention of participants at 12 weeks follow-up, app use, perceived usefulness and usability of the app, as well as reasons for dropout in participants who will have discontinued participation (assessed via email).

As exploratory outcomes, we will assess knowledge of risk and protective factors for dementia (dementia literacy), change in dementia risk profiles (risk/protective factors exhibited by participants), perceived barriers towards and motivation for behaviour change. Changes in dementia risk profiles are expected primarily in behavioural and symptom-related components of the LIBRA-score, including nutrition, physical activity, alcohol consumption, smoking, cognitive and social activity, sleep and depressive symptoms. Chronic conditions like, for example, diabetes or coronary heart disease are not expected to resolve during the 12-week intervention and will be used primarily to characterise participants’ risk profiles. The same instruments will be administered both at baseline and follow-up to assess changes in the LIBRA-score and its individual components, based on participants’ self-report. A full description of outcomes and respective assessments applied is given in table 1.

**Table 1:** Outcomes and assessments to be used in the course of the pilot study

Construct	Instrument	BL	FU
Key feasibility outcomes			
Success and feasibility of recruitment	Information on: numbers of individuals (1) interested in the study, (2) signed informed consent, (3) eligible for participation; time necessary for recruitment; response to each recruitment channel; diversity of participants; time and resources spent on different recruitment channels; time from initial contact to enrolment, collected at study centre, standardised forms	x	
Retention rate	Rate of participants completing the follow-up assessment		x
App use	Meta-data on (1) downloads of the app, (2) interactions with the app, (3) app modules used, (4) topics chosen to address, (5) number of notifications received		x
Usability	German mHealth App Usability Questionnaire – Short Version (G-MAUQ^44^)		x
Usefulness	Technology Acceptance Model Questionnaire (TAM^45^)		x
Non-use of the app, dropout	Standardised questions on reasons for non-use of the app or leaving the study in participants who will have dropped out		x
Exploratory outcomes			
Changes in dementia risk profile	LIBRA-score, comprising information on: *Nutrition*: Mediterranean Diet Adherence Screener (MEDAS^46^); Dietary Fat and Free Sugar – Short Questionnaire (DFS^47^) *Physical activity*: International Physical Activity Questionnaire (IPAQ^48^) *Cognitive activity*: ‘Leisure Time’-scale of the Cognitive Reserve Index Questionnaire (CRIq^49^) *Alcohol consumption, smoking*: standardised questionnaire^50^ Hypercholesterolaemia, diabetes, hypertension, obesity, coronary heart disease, chronic kidney disease: standardised questionnaire^51^ *Depression*: Patient Health Questionnaire (PHQ-9^52^) *Social activity*: Lubben Social Network Scale (LSNS-6^53^); loneliness (UCLA Loneliness Scale^54^) *Hearing impairment*: Mini-Audio-Test (MAT^55^); possession and use of hearing aids *Sleep*: Athens Insomnia Scale for non-clinical application (AIS-NCA^56^)	x	x
Dementia literacy	Standardised questionnaire on knowledge of modifiable risk factors for dementia^19 20^	x	x
Health-related quality of life	EuroQol Visual Analogue Scale (EQ-VAS^57^)	x	x
Self-efficacy	General self-efficacy^58^; domain-specific self-efficacy (LIBRA-factors nutrition, physical activity, social activity, cognitive activity, smoking cessation, alcohol consumption^59^)	x	x
Motivation for behaviour change	Standardised questionnaire, as applied in Zülke *et al*^60^; reason for participation (standardised forms)	x	x
Sociodemographics	Standardised questionnaire (age, sex, education, occupational status, living situation, marital status)	x	

BL, baseline; FU, follow-up; LIBRA, LIfestyle for BRAin Health.

### Sample size

As our study is a pilot study applying a pre-post design, a power analysis to determine sample size was not indicated. To assess use and usefulness of the MyBraincoach app, a sample size of n=75 is deemed sufficient to obtain data of n=60 participants for analyses at follow-up, expecting a dropout rate of 20%.

### Data management

Information necessary to assess success and feasibility of recruitment will be assessed by the research team at the study centre, using standardised custom-made forms. Download and use of the app, including information on specific app features and number of interactions with the app, will be assessed using meta-data, provided by the web-design agency Indicia Betawerk, responsible for development and hosting of the app. Baseline and follow-up assessments will be conducted online using Limesurvey software. Secured hyperlinks to the baseline and follow-up assessment will be sent via email to all individuals who provided online informed consent to participate.

Safeguards will be implemented to prevent unauthorised changes of data (passwords, access rights), and only researchers entrusted with data handling, reimbursement of participants and analyses within the project will have access to the data. Source documents and data management procedures will be maintained readily available. Quality of data (eg, data from online assessments, documents regarding study and participant management, etc) will be monitored continuously. Data protection laws, such as the European General Data Protection Regulation, will be followed and every person having or requesting access to the data is obliged to confidentiality. All personal information of participants will only be documented for the duration of the study and will be saved separately from meta-data and data collected at the baseline and follow-up assessments, complying with data protection laws. Account data needed for reimbursement of participants will be deleted immediately on completion of reimbursement. Personal identification data of participants will be saved on a central server restricted by password access after assigning a randomised pseudonym ID to each participant. All analyses conducted within the project will solely use de-identified, pseudonymised data.

Meta-data, for example, on number of app downloads, logins and interactions with the app, are stored on a secured server in the Netherlands. Data are transferred using a Secure Sockets Layer certificate and will only be stored in a pseudonymised fashion to ensure participant anonymity. On signing the informed consent, participants will further be informed of the modes and purposes of data collection, storage and usage, as well as their rights to demand correction or deletion of their data.

### Data analysis plan

#### Quantitative analyses

All acquired data will be examined for potential inconsistencies and missing values. Drop-out analyses will assess potential systematic biases between participants who complete the follow-up assessment 12 weeks after baseline and non-completers, using χ² and two-sample t-tests, as appropriate. Success and feasibility of recruitment, retention of participants, use and usability of the app as well as reasons for dropouts will be described using means (SDs) and percentages, as appropriate. Changes in LIBRA, dementia literacy, health-related quality of life, self-efficacy and motivation for behaviour change will be described using dependent samples t-tests. The significance level of all analyses will be set to p<0.05 (two-tailed). Analyses will be conducted using Stata/SE V.16.0. Results will be reported according to the extended guidelines of the CONSORT statement for pilot and feasibility trials.^30^

#### Qualitative analyses

A single remote, video-based focus group targeting n=8 participants will be conducted as a formative supplement to the quantitative feasibility assessment. The focus group is intended to provide exploratory insights into app use and usability and identify suggestions for intervention refinement, rather than to achieve thematic saturation. A researcher experienced in group interviews will moderate the focus group (expected duration: 90–120 min), which will be conducted using appropriate software (eg, Zoom). The interview guide will be designed following the guidelines for problem-centred interviews,^42^ a semi-structured type of interview aiming to provide impulses for discussion and free narration of participants. Topics to be covered include previous experience with eHealth/mHealth tools, previous knowledge of lifestyle factors relevant for dementia risk reduction, perception of app-design, use and usability of specific app-features, evaluation of app content and suggestions for improvement, using open-ended questions. The focus group will be video-recorded and transcribed fully by an experienced transcribing service.

Content of the focus group will be analysed using structured content analyses.^43^ First, categories will be built inductively to analyse interview transcripts (coding scheme). Second, transcripts and derived categories will be entered into MAXQDA software. Finally, blocks of transcripts will be assigned to the coding scheme. Results will be processed quantitatively in MAXQDA. Sociodemographic information of focus group participants (age, sex, education) will be analysed descriptively using Stata.

### Patient and public involvement

Usability testing sessions, particularly targeting people with lower degrees of education and with disabilities, are an integral part of the study and will be conducted before the pilot study to ensure accessibility of the intervention across various backgrounds of participants and gather participant feedback. Further, we aim to gain a better understanding of user experiences with the app and gather potential suggestions for improvement by means of a moderated focus group following completion of the pilot study. This approach will increase participant involvement in the design of the intervention to be tested in a subsequent confirmatory RCT, including implementation of suggested changes and adaptations to the app, if feasible.

## Ethics and dissemination

###  Ethical approval

The study was approved by the Ethics Committee of the Medical Faculty of Leipzig University (ref.: 476/22-ek).

### Consent to participate

All participants in the pilot study will provide online informed consent to participate prior to participation, using an electronic Transport Layer Security-encrypted consent form. Potential participants will be informed about details of the study, voluntariness of participation, the option of discontinuing participation at any time and details on data protection. Usability testers will sign a written informed consent form prior to the usability testing session. Participants having agreed to be re-contacted after the pilot study and willing to participate in the focus group will sign an additional online informed consent prior to participation in the focus group.

### Adverse events

We will investigate a mHealth intervention targeting general lifestyle behaviour to reduce dementia risk. By providing individuals with personalised feedback on their individual risk profile for dementia, the intervention might raise fear or concern among participants to some degree. However, this risk is deemed very low, as participants will be informed about the study aims before consenting to participate, that is, a general interest and willingness to engage with the topic can be assumed. As the intervention targets everyday behaviour beneficial for a healthy lifestyle, is aimed at generally healthy individuals and advice within the app will suggest consulting a GP in case of uncertainty, potential harm for participants is deemed negligible. Potential adverse events occurring during the pilot study will be assessed, regardless of a causal link to the study. Participants will be able to document any problems arising during use of the study within the app. Contact details of the research team will be placed prominently within the app and participants will be asked to contact the research team whenever encountering problems regarding study participation and/or app use.

### Dissemination plan

The results of this study will be disseminated at conferences and in publications in peer-reviewed scientific journals, published open access where possible. All reported data will not allow for inference to study participants, but will be reported anonymously. Study design and progress, as well as results, will also be disseminated using social media (eg, X, LinkedIn) to inform the general public. The participant-level dataset will not be made publicly available due to data protection and consent restrictions.
