# Supplementary material for: Feasibility, use and usability of an adapted mHealth tool for dementia risk reduction among middle-aged and older adults in Germany: protocol for a non-randomised pilot study
Source: BMJ Open. 2026 Sep 16;16(9):e117886. doi: 10.1136/bmjopen-2026-117886 (PMC13583851; doi:10.1136/bmjopen-2026-117886)
Supplement: Supplementary file 1 [file bmjopen-2026-117886.reviewer_comments.pdf]

PEER REVIEW HISTORY

BMJ Open publishes all reviews undertaken for accepted manuscripts. Reviewers are asked to complete a checklist review form and are provided with free text boxes to elaborate on their assessment. These free text comments are reproduced below.

ARTICLE DETAILS

Title (Provisional)

Feasibility, use and usability of an adapted mHealth tool for dementia risk reduction among middle-aged and older adults in Germany – protocol for a non-randomized pilot study

Authors

Zülke, Andrea E; Luppa, Melanie; Deckers, Kay; Köhler , Sebastian; Riedel-Heller, Steffi G.

VERSION 1 - REVIEW

**Reviewer**	**1**
**Name**	**Gunasekaran, Venugopalan**
**Affiliation**	**Jawaharlal Institute of Postgraduate Medical Education, Geriatric Medicine**
**Date**	**11-Jun-2026**
**COI**	**None**

I appreciate the authors for a detailed well written protocol.

The title is more reflective of the secondary outcome. The primary outcome of checking the feasibility / usability of an existing app in German population is not reflected in the title. A change can be considered if possible

---

**Reviewer**	**2**
**Name**	**Wang, Huali**
**Affiliation**	**Peking University Sixth Hospital, Clinical Research Division**
**Date**	**06-Aug-2026**
**COI**	**None**

Following the launch of the WHO updated guideline on risk reductrion of cognitive decline and dementia, the sutdy is timely to implement the guideline in real-world settings. However, there are a few concerns to be addressed for further improvement of the article.

1. Though the study has great potential to promote brain health in community; however, the research gap and novelty of the study to be conducted among German population should be more explicitly articulated. The current statement “Respective studies from Germany are lacking so far” is too weak. It would be more informative if the introduction could be strengthened by specifying how this work will bring unique contribution to the field of dementia prevention and what novel knowledge this work will advance for implementing the updated guideline.

2. Several primary objectives are set for this work. Although the sample size estimation is not specifically indicated with targeting primary objective, it would be scientifically sounding if the primary objective could be more focused rather than widespread.

3. Concerning the secondary objectives, how to measure the changes in dementia risk profiles during the study period. The APP includes recommendation for modifying 15 risk factors. As widely understood, several risk factors, such as diabetes, hypertention, and depression, may not improve in such a short study period. It would be necessary to highlight what risk profiles are expected to change and how to measure or monitor the change over time.

4. Please clarify whether those with relatively low risk profile will be recruited as the change of risk profiles in this group of people may not be sensitive to be detected.

5. Although it is a good design to include qualitative interviews in the study design, the rationale and methodology of only conducting one single focus group (n=8)? What criteria are used to estimate the number of focus groups and the size of each focus group?

VERSION 1 - AUTHOR RESPONSE

Comment no.	Comments	Response	Amendments
	**Reviewer 1**		
1	I appreciate the authors for a detailed well written protocol.	We thank the reviewer for their kind acknowledgement of our manuscript!	
2	The title is more reflective of the secondary outcome. The primary outcome of checking the feasibility / usability of an existing app in German population is not reflected in the title. A change can be considered if possible	We agree with the reviewer that the original title did not adequately reflect the focus of the study. The title was revised accordingly.	Revised title: “Feasibility, use and usability of an adapted mHealth tool for dementia risk reduction among middle-aged and older adults in Germany – protocol for a non-randomized pilot study”

	Reviewer 2		
1	Following the launch of the WHO updated guideline on risk reduction of cognitive decline and dementia, the study is timely to implement the guideline in real-world settings. However, there are a few concerns to be addressed for further improvement of the article.	We thank the reviewer for their kind acknowledgement of our manuscript!	
2	Though the study has great potential to promote brain health in community; however, the research gap and novelty of the study to be conducted among German population should be more explicitly articulated. The current statement "Respective studies from Germany are lacking so far" is too weak. It would be more informative if the introduction could be strengthened by specifying how this work will bring unique contribution to the field of dementia prevention and what novel knowledge this work will advance for implementing the updated guideline.	Following the reviewer's suggestion, we further point out the current research gap and respective contribution of the present study. We further added a reference to the updated WHO guidelines on risk reduction of cognitive decline and dementia.	Lines 110ff: "The recently updated World Health Organization (WHO) guidelines on risk reduction of cognitive decline and dementia emphasize the potential of tailored multidomain interventions across the life course, targeting several risk factors for dementia simultaneously. This includes interventions targeting depression, sleep, hearing loss and social isolation. The WHO further emphasizes the need for culturally tailored and context-specific interventions, delivered in-person or digitally, to increase accessibility and reach. To date, implementation research addressing feasibility and scalability of respective interventions remains limited."<br><br>Lines 135f: "However, it remains unclear whether a culturally tailored multidomain mHealth intervention can be successfully implemented and accepted in a German population."<br><br>Lines 164ff: "The present study aims to provide context-specific evidence on recruitment and retention, patterns of app engagement, perceived usefulness and

			usability and accessibility of an adapted version of the app in a German setting. By combining a quantitative feasibility assessment with qualitative user feedback, it will provide implementation knowledge required to refine the intervention and inform the design of a subsequent randomized controlled trial.”
3	Several primary objectives are set for this work. Although the sample size estimation is not specifically indicated with targeting primary objective, it would be scientifically sounding if the primary objective could be more focused rather than widespread.	We thank the reviewer for this comment. We revised the manuscript to specify one overarching primary objective (assessment of feasibility and acceptability), while distinguishing key feasibility outcomes from exploratory outcomes. As this pilot study is not designed to assess effectiveness, no single primary endpoint was specified, and the sample-size section now clarifies that n = 75 was selected to estimate feasibility parameters rather than based on a formal power calculation.	Abstract, lines 42f: “**Key feasibility** outcomes include feasibility and success of recruitment, use, and usability of the app. **Exploratory** outcomes include changes in dementia risk profiles, dementia literacy, health-related quality of life, self-efficacy, and motivation for behavior change.”<br><br>Lines 139ff: Feasibility and acceptability will be evaluated in terms of:<br>• feasibility of recruitment<br>• success rate of retention of participants at 12-weeks follow-up assessment<br>• usage (patterns) of the app<br>• perceived use and usability of the app<br>• reasons for non-use and study dropout.<br>Further exploratory objectives are:<br>• To investigate change in knowledge of risk and protective factors for dementia<br>• To describe changes in dementia risk profiles at 12-weeks follow up<br>• To assess changes in self-efficacy, motivation for behavior change, and health-related quality of life

			• To gain exploratory insights into participants' perception of the app by means of a moderated focus group."<br>Lines 305ff: "As this pilot study is not designed to assess intervention effectiveness, no single primary endpoint was specified. The primary objective is to assess the feasibility and acceptability of the adapted MyBraincoach app and the associated study procedures. No single primary endpoint was specified, as the study is not designed to assess intervention effectiveness. Key feasibility outcomes include..."<br>Line 313: "As exploratory outcomes, we will assess..."<br>Table 1: "***Key feasibility** outcomes"; "**Exploratory** outcomes"*
4	Concerning the secondary objectives, how to measure the changes in dementia risk profiles during the study period. The APP includes recommendation for modifying 15 risk factors. As widely understood, several risk factors, such as diabetes, hypertension, and depression, may not improve in such a short study period. It would be necessary to highlight what risk profiles are expected to change and how to measure or monitor the change over time.	We thank the reviewer for pointing this out. The revised manuscript describes more clearly which modifiable risk factors are expected to be sensitive to change in the applied study period, and how changes will be assessed.	Lines 316ff: "Changes in dementia risk profiles are expected primarily in behavioral and symptom-related components of the LIBRA-score, including nutrition, physical activity, alcohol consumption, smoking, cognitive and social activity, sleep and depressive symptoms. Chronic conditions like, e.g., diabetes or coronary heart disease are not expected to resolve during the 12-week intervention and will be used primarily to characterize participants' risk profiles. The same instruments will be administered both at baseline and follow-up to assess changes in the LIBRA-score and its individual components, based on participants' self-report."
5	Please clarify whether those with relatively low risk profile will be recruited	We agree with the reviewer that room for improvement in individuals with relatively	Lines 79ff: "Participants will be included irrespective of their baseline dementia risk to

	as the change of risk profiles in this group of people may not be sensitive to be detected.	few risk factors for dementia might be limited, which is now acknowledged as a limitation. However, since the primary aim is to assess feasibility, use and usability of the app in a community-based population, rather than intervention effectiveness, no minimum number of risk factors/minimum LIBRA-score is required, which was specified in the methods-section.<br>Participants with low-risk profiles may still benefit from knowledge-based content and recommendations to maintain protective behaviors.	enhance the applicability of the findings to the general population, which may limit detectable changes in dementia risk profiles due to potential floor effects."<br>Lines 188ff: "No minimum number of modifiable risk factors will be required for study participation, and individuals with few or no risk factors will remain eligible, as the intervention also aims at improving knowledge about the links between lifestyle and brain health and reinforce the maintenance of protective health behaviors."<br>Lines 260ff: "Preferably, participants will select a factor indicating room for improvement. However, participants with limited or no room for improvement may freely choose any lifestyle factor or health condition covered by the app."
6	Although it is a good design to include qualitative interviews in the study design, the rationale and methodology of only conducting one single focus group (n=8)? What criteria are used to estimate the number of focus groups and the size of each focus group?	The qualitative component is intended as a formative supplement to the quantitative feasibility study, rather than as a stand-alone qualitative study aiming to achieve thematic saturation. We therefore considered one focus group targeting eight participants sufficient to explore key usability issues and assess suggestions for intervention refinement, while allowing all participants adequate opportunity to contribute. Participant selection will consider level of app engagement, education and gender, subject to the composition and availability of participants consenting to re-contact. Findings will be interpreted as exploratory and will not be assumed to represent the full range of user experiences.	Lines 225ff: "Participants will be purposively selected from those consenting to be re-contacted. Subject to participant availability, selection will aim to include individuals with different levels of app engagement, varied educational and sex backgrounds."<br><br>Lines 377ff: "A single remote, video-based focus group targeting n = 8 participants will be conducted as a formative supplement to the quantitative feasibility assessment. The focus group is intended to provide exploratory insights into app use and usability and identify suggestions for intervention refinement, rather than to achieve thematic saturation."

VERSION 2 - REVIEW

**Reviewer**	**2**
**Name**	**Wang, Huali**
**Affiliation**	**Peking University Sixth Hospital, Clinical Research Division**
**Date**	**01-Sep-2026**
**COI**	

The reviewers' concerns have been addressed adequately.
